# The Atlanta Medical College—Its Prospects

**Published:** 1867-04

**Authors:** 


### EDITORIAL AND MISCELLANEOUS

### THE ATLANTA MEDICAL COLLEGE—ITS PROSPECTS.

This Journal has, in former times, often taken occasion to allude to the status of the College, and predict its future. We have given its condition and success from facts coming under our knowledge, and what it is afterwards expected to accomplish, in part from its former history, and also from the legitimate results known to follow certain natural causes.

The season selected, at the establishment of the College, for the Course of Lectures, was a fortunate one; and shows the foresight of him who suggested and advocated till adopted, the plan of holding the regular Session during the summer months. His voice, though still in death before the first class assembled, was heard in emphatic strains at the meeting in 1854, when this system was adopted. And while we yet deeply lament his loss to the profession, we feel still more keenly his loss in the School, to whose sagacity and wise counsels it is to a large extent indebted for its subsequent prosperity. The name of Dr. J. M. Gordon will be ever held in sad remembrance by those associated with him at the foundation of the Institution. And while this fact was alluded to years ago, in the Journal, we cannot refrain now from offering again this slight tribute to his memory.

Certain natural causes lead to the same results now as in 1854. That reasoning which decided the system of instruction pursued by this College, has been urged again and again as evidence of its permanency and continued prosperity. The great central locality, in the midst of ten or twelve States, in which no regular course of Medical Lectures is held, except in winter; the accessibility, healthfulness, and good water,—are some of the reasons why Atlanta is a suitable location for a Medical College, and why the Summer months are more appropriate.

The exploded idea that dissections can not be profitably and conveniently prosecuted during Summer, is "among the things that were." It is only necessary to find any member of the eight Classes that have demonstrated the fact in the dissecting room here, to get evidence on this subject.

Heretofore, the only objection that could, with any plausibility, be urged against Atlanta as the very best point in the surrounding States, to build up a great and prosperous Institution was the idea, that sufficient material for clinical instruction could not be made available. Experience, at least since the late war, has proven the absurdity of this objection, as the Class in attendance at the last session of this Institution, can well testify.

With an Hospital in the College yard, having an average of one hundred and fifty patients, and a daily Dispensary in the College building, fed from a population of from eight to ten thousand *freedmen*, and, perhaps, one thousand poor whites, we have abundant material for clinical instruction—more certainly than can be presented to any class with profit. We say with profit, as a Medical Class must, necessarily, devote much of their time to other departments included in the regular curriculum.

Notwithstanding the vaunted clinical advantages claimed for the Schools in the large Cities, we do not hesitate to assert, that the clinical material is as abundant in Atlanta as that presented at any one of the College clinics in Philadelphia,—the heretofore center of Medical education.

It is true, that these Colleges can boast of their extensive City Hospitals,—as the Blockly, Philadelphia Hospital, etc.; but all know, who have spent a winter in that City, that the mass of students in attendance, rely exclusively upon the College Dispensaries for clinical instruction.

